# Identification of flowering-time genes in mast flowering plants using *De Novo* transcriptomic analysis

**DOI:** 10.1371/journal.pone.0216267

**Published:** 2019-08-14

**Authors:** Samarth, Robyn Lee, Jiancheng Song, Richard C. Macknight, Paula E. Jameson

**Affiliations:** 1 School of Biological Sciences, University of Canterbury, Christchurch, New Zealand; 2 Department of Biochemistry, University of Otago, Dunedin, New Zealand; 3 School of Life Sciences, Yantai University, Yantai, China; Youngstown State University, UNITED STATES

## Abstract

Mast flowering is synchronised highly variable flowering by a population of perennial plants over a wide geographical area. High seeding years are seen as a threat to native and endangered species due to high predator density caused by the abundance of seed. An understanding of the molecular pathways that influence masting behaviour in plants could provide better prediction of a forthcoming masting season and enable conservation strategies to be deployed. The goal of this study was to identify candidate flowering genes that might be involved in regulating mast flowering. To achieve this, high-throughput large-scale RNA-sequencing was performed on two masting plant species, *Celmisia lyallii* (Asteraceae), and *Chionochloa pallens* (Poaceae) to develop a reference transcriptome for functional and molecular analysis. An average total of 33 million 150 base-paired reads, for both species, were assembled using the Trinity pipeline, resulting in 151,803 and 348,649 transcripts respectively for *C*. *lyallii* and *C*. *pallens*. For both species, about 56% of the unigenes were annotated with gene descriptions to known proteins followed by Gene Ontology analysis, categorising them on the basis of putative biological processes, molecular function, and cellular localization. A total of 543 transcripts from *C*. *lyallii* and 470 transcripts from *C*. *pallens* were also mapped to unique flowering-time proteins identified in *Arabidopsis thaliana*, suggesting the conservation of the flowering network in these wild alpine plants growing in natural field conditions. Expression analysis of several selected homologous flowering-pathway genes showed seasonal and photoperiodic variations. These genes can further be analysed to understand why seasonal cues, such as the increasing photoperiod in spring, that triggers the annual flowering of most plants, are insufficient to always trigger flowering in masting plants and to uncover the molecular basis of how additional cues (such as temperature during the previous growing seasons) then determines flowering in mast years.

## Introduction

RNA-sequencing (RNA-seq) has enabled the expansion of molecular biology into the fields of ecology and evolution [1]. Researchers can analyse not only differentially expressed genes but also identify splice variants, rare transcripts, and novel small RNA targets in plants [2–4]. The use of ‘ecological transcriptomes’ has increased rapidly as they enable the study of non-model plant species growing in naturally fluctuating environments, especially as sequencing and assembling large genomic datasets is complex and expensive [5]. A search of the Web of Science (Thomson Reuters) database with the term RNA-seq/RNAseq resulted in 2,147 articles published between 2010–2019 with 114 articles already published in 2019 in the plant sciences (S1 Fig). Refining the search to the field of ecology and evolution returned 734 articles published in the last five years. The application of next-generation sequencing has led to the discovery of novel genes and associated molecular markers from non-model plants which have been used to improve breeding efficiency, quality and nutritional value of agronomic crops [6, 7].

A defined flowering time is a critical ecological trait necessary for a plant to achieve its reproductive potential [8]. Flowering at the appropriate time enables greater seed set and distribution, and the avoidance of abiotic or biotic stresses prevailing in the environment [9]. At the population level, the timing can affect the fitness of the individual species along with the prevailing competition in an ecosystem [10]. Synchronisation of flowering with the growth and development cycles of insects and animal pollinators will allow greater pollination success which, in turn, will affect the species distribution as well as species richness in a geographical area [11].

A detailed knowledge of the molecular mechanisms controlling flowering time has been acquired for model annual plants, such as *Arabidopsis thaliana* (arabidopsis) and rice, which reproduce once and die [12]. The mechanisms controlling flowering are broadly conserved in all plants, with inductive environmental cues, commonly day length and low temperature, resulting in the expression of an *FT* gene that encodes a floral signal molecule that is produced in the leaves and transported to the apex where it triggers flowering.

Unlike annual plants, flowering in perennials is a continuous process of alternation between vegetative and reproductive phases [13]. Previous reports have identified a strong correlation between expression of *FT* induced by flowering promoters and onset of the reproductive phase change [14]. Modulation of expression changes in *FT* in response to seasonal fluctuations has helped unravel how flowering is controlled in perennial plants [15]. Rather than just relying on predictable seasonal cues to determine the timing of reproduction, some species show supra-annual synchronous flowering within a plant population at irregular intervals. These plant species are known as masting plants with high, fluctuating seed production over years [16]. This unique phenomenon has supra-annual patterns caused by changes in seasonal temperatures over two successive years as hypothesized from long-term phenological data [17]. Consequently, in masting species, there must be an added layer of regulation where the annual seasonal cues are not sufficient to trigger flowering and additional cues are necessary. A first step to discover the molecular basis of how flowering is controlled in masting species is to use RNA-seq to identify the genes known to control flowering in other species.

Masting provides selective benefits to the plant population involving predator satiation, greater pollination efficiency, better seed dispersal, and food for native birds and mammals [18]. However, it also leads to an explosive increase in the population of predatory animals feeding on endangered and endemic birds in Aotearoa New Zealand. Masting events are responsible for significant decreases in the population of kiwi, kakapo and kea due to the attacks by huge populations of rats and stoats [16, 19]. Therefore, understanding the molecular causative mechanism of a masting event can supplement the phenological and mathematical datasets for systematic conservation planning within the natural ecosystem.

Synthesising molecular knowledge about masting can also benefit ecological and evolutionary studies enabling inferences to be drawn from the phenological data available from diverse geographical habitats. Molecular knowledge can facilitate forecasting of future changes in masting behaviour and underpin assessment of how future changes in natural conditions may lead to molecular adaptation of flowering-time genes and associated regulatory mechanisms affecting the masting pattern [6].

In this study, we used RNA-sequencing and gene expression analysis to identify the flowering-time genes that may be involved in mast flowering in *Chionochloa pallens* and *Celmisia lyallii*. *C*. *lyallii* (Asteraceae) and *C*. *pallens* (Poaceae) are two alpine perennial plants exhibiting strong masting behaviour [20]. These two plant species provide an excellent model for the molecular study of masting as there are extensive field study and modelling data available to describe the mast flowering pattern [17]. Expression analyses of the flowering-time marker genes may help to unravel the threshold and accurate timing of the reception of the flowering cue in plants associated with natural environmental conditions. Molecular studies can further supplement the ecological data to better understand the flowering phenology of masting plants.

## Materials and methods

### Plant material

Samples were collected from the natural site of *C*. *pallens* at 1070 m and *C*. *lyallii* at 1350 m of Mt Hutt (43.4717° S, 171.5264° E) during late summer (15^th^ of March 2015 for *C*. *pallens* and 22^nd^ of March 2016 for *C*. *lyallii*) for transcriptome analysis. The Department of Conservation permit number 40225-FLO allowed for the collection of the plants used in this study. Three replicates from four different tissues (roots, mature, old and young leaves) from *C*. *lyallii* were collected for RNA-sequencing. These replicates were pooled together to generate a single transcriptome library for *C*. *lyallii*. For *C*. *pallens*, three independent replicates each comprising of three separate leaf and apical meristem tissues were collected during mid-morning (11:00 AM) for sequencing analysis (six different libraries in total). Leaf tissue samples were also collected for both species from their respective field sites throughout the year. Samples were stored at -80°C until further processing.

### RNA extraction and *de novo assembly*

Total RNA was extracted from the *C*. *lyallii* (leaves and root samples) and *C*. *pallens* samples (leaf and meristematic tissue) using Qiagen Plant RNA extraction kit. The extracted RNA was DNase I digested and checked for quality using a Bioanalyser 2100 (Agilent Technologies). RNA isolated from each of the samples had a RIN greater than 7 and an rRNA ratio greater than 1, both being essential parameters for RNA quality prior to sequencing. cDNA libraries were prepared using an Illumina TruSeq kit 2.0 followed by paired-end sequencing on the Illumina HiSeq 2500 platform [21]. Sequencing for *C*. *lyallii* was done at Macrogen Inc. (South Korea), while sequencing for *C*. *pallens* was carried out at NZGL (Dunedin, New Zealand). RNA isolated from the leaf tissues and roots of *C*. *lyallii* were pooled together to generate one cDNA library. For *C*. *pallens*, separate libraries were prepared for the three biological replicates of leaf and apical meristem tissue.

The 150–base pair paired-end reads that were generated were checked for quality. Bases with a quality score of less than 30 and reads containing fewer than 20 bases were removed using the fastq quality trimmer program. Adapters were removed using the Trimmomatic program with default parameters [22]. Quality of the raw data generated from both species was again analysed using the Fastqc program [23]. Cleaned reads were then assembled together into a single reference transcriptome assembly using the Trinity pipeline with default parameters [24]. The combined *de novo* assembly is referred to as the ‘*C*. *lyallii* draft transcriptome’ and ‘*C*. *pallens* draft transcriptome’, respectively. These transcriptomic datasets were used in all the analyses reported in this paper. All the sequences in these datasets have been deposited at NCBI as sequence read archive files (Accession numbers: SRR9595649, SRR9595648, SRR9595651, SRR9595650, SRR9595647, SRR9595646, SRR9591156). The Trinitystats.pl script was used to assess the completeness of the assembled transcriptome based on the assembly statistics. Additionally, the eukaryotic Benchmarking Universal Single-copy Orthologs (BUSCOs) dataset was compared with our assembled transcriptome datasets to identify single gene copy orthologous sequences [25].

### Functional annotation of the assembled transcriptome

Open Reading Frames (ORFs) with a length of at least 100 amino acids were predicted using the Transdecoder script for both transcriptome datasets. Standalone BLAST was used to perform sequence similarity searches to annotate the obtained unigenes. The transcriptomic assembly was compared against publicly available protein databases of arabidopsis, *Helianthus annuus* (sunflower), and *Chrysanthemum* (chrysanthemum) for *C*. *lyallii*, and arabidopsis, *Zea mays* (maize), *Oryza sativa* (rice) for *C*. *pallens* using blastx with an E-value cut off of 10^−3^. The predicted peptides annotated from the blastx searches were again filtered using a cut-off value of 50% identity match and 60% query coverage for stringent gene annotation. Only unique genes identified from the similarity search by blastx were then used for Gene Ontology (GO) analysis. GO analysis was performed using PANTHER based on the search output from the BLAST results to classify unigenes into molecular function, biological processes, and cellular component categories [26]. The unigenes obtained were also blasted against the NCBI-nr, Swiss-Prot, PlantTF databases and Clusters of Orthologous Groups of proteins (COG) (E-value ≤ 1.0E-5) to further annotate the unigenes. Unigenes aligned to the COG database were categorised into their possible functions. Unigenes were also mapped to the KEGG database with an E-value threshold of 10^−5^ to explore pathway mapping and function in both species [27].

### Differential expression analysis

Transcript abundance estimation was performed between leaf and apical meristem tissue from *C*. *pallens* using Trinity-inbuilt script. A transcript abundance matrix was generated using bowtie 2.0 by aligning the trimmed reads from each sample to the ‘*C*. *pallens* draft transcriptome’. The DESeq2 package in R was used to calculate the differential transcript expression between different samples based on the generated transcript abundance matrix [28]. Differentially expressed unigenes were mapped against the Flowering-Interactive Database (FLOR-ID) to identify floral genes which may be involved in the activation of the flowering process in *C*. *pallens*. For *C*. *lyallii*, RNAseq was performed using a single sample of pooled RNA and, consequently, it was not possible to perform a differential expression analysis.

### Identification of flowering-time genes

The assembled transcriptomes were imported and maintained in the form of a collective database on the CLC genomics workbench and are referred to as the ‘*C*. *lyallii* database’ and ‘*C*. *pallens* database’ respectively. Flowering-time gene sequences from the FLOR-ID database of arabidopsis were used as the reference sequences to identify the corresponding orthologs in *C*. *lyallii* and *C*. *pallens*. The assembled transcriptomes were BLAST searched against the FLOR-ID [29] database with E-value 1e-5 and 70% protein identity. Additional, similar homologous protein sequences of flowering-time genes from sunflower and rice were also BLAST searched against the assembled transcriptomes for accurate characterization of flowering-time genes in the *C*. *lyallii* and *C*. *pallens* databases using tblastn. The sequence with the highest score and lowest e-value was selected as a putative target sequence along with the results from the search against the arabidopsis flowering database.

### Reverse transcriptase quantitative PCR (RT-qPCR)

Samples collected throughout the year from the field sites were used to analyse seasonal expression of selected flowering-time genes. Validation of the differential expression analysis was also done using RT-qPCR. Total RNA was isolated using a Plant RNA extraction kit from Qiagen as mentioned above. cDNA was synthesized using 1 μg of RNA and home-made reverse transcription mix (50 μM oligodT, 50 μM random hexamers, 10 mM dNTPs, 5X first-stand buffer, 0.1 M DTT, 40 U/μl RNaseOUT, and 200 U/μl Superscript III reverse transcriptase) [30]. Real-time quantitative PCR was carried out using 15 μL SYBR reaction mixture (Kapa Biosystems) in a RotorGene Q cycler (Qiagen). Relative gene expression levels were calculated using the 2^- ΔΔCt^ method [30, 31]. No-template controls and negative reverse transcriptase reactions were also set up alongside the qPCR batch to confirm the absence of genomic DNA and other contaminants in the sample. Protein pyrophosphatase 2A and GAPDH for *C*. *lyallii*, Expressed protein and Tumour homolog protein for *C*. *pallens* were used as the reference genes [32]. The list of primer sequences used in the present investigation is given in S1 Table.

## Results and discussion

### *De novo* transcriptome assembly

The first aim of this study was to obtain a catalogue of genes expressed in the two masting species, *C*. *lyallii* and *C*. *pallens*, to aid the identification of genes involved in ecologically significant traits, in particular the genes controlling flowering. For *C*. *lyallii*, samples were chosen from leaves and roots to build a complete reference transcriptome. Leaf and apical meristem samples from *C*. *pallens* were collected to build a reference transcriptome and separate libraries for each of the biological replicates were prepared in order to perform differential expression analysis. Differential expression analysis between leaf and apical meristem was carried out to enhance our understanding of the genes involved in the regulation of flowering in *C*. *pallens*.

cDNA libraries, developed from leaves and root samples of *C*. *lyallii* and leaves and apical meristem samples of *C*. *pallens* were sequenced using Illumina HiSeq. The *C*. *lyallii* and *C*. *pallens* draft transcriptomes consisted of about 151,803 and 348, 649 transcripts, respectively (Table 1). The assembled transcriptomes were used in all further analyses. From these assembled transcriptomes, 92, 805 and 152, 250 transcripts were predicted to encode peptides which were at least 100 amino acids long for *C*. *lyallii* and *C*. *pallens*, respectively. The high number of Trinity transcripts could be due to the high ploidy level in *C*. *pallens* and the massive genome size for *C*. *lyallii* (2n = 108) which can contribute to adaptability to the complex environment from which the plant samples were taken.

**Table 1:** Assembly statistics for the *de novo* transcriptome.

	*Celmisia lyallii*	*Chionochloa pallens*
**Total no of reads**	37,147,626	29,282,017
**High quality reads**	37,030,237	29,281,644
**Cleaning rate**	99.68399327	99.98
**Total Trinity genes**	66,496	210,932
**Total Trinity transcripts**	151,803	348,649
**% GC**	40	48.68
**N50**	1,524	795
**Median contig length**	780	506
**Average contig length**	1,083	705
**Coverage (X)**	33	18
**Total assembled bases**	164,343,983	245,893,094

Many genomic features, including gene length, gene density, meiotic recombination and gene expression, have often been shown to be associated with the GC content of an organism. The GC content is highly variable among distinct species and is usually more pronounced in heterogeneous populations [33]. Eukaryotes have a GC content varying between 20 and 60%. [34]. The draft transcriptome of *C*. *lyallii* has a 40% GC content which is consistent with the other dicot plant species [35]. In contrast, *C*. *pallens* has 48% GC content suggesting it may be a wild ancestral member of the Poaceae family [36]. A high GC content is often associated with high tolerance/adaptive capability. This could explain why *C*. *pallens* is widespread across New Zealand and adapted to varied environmental conditions.

### BUSCO analysis

Benchmarking Universal Single Copy Orthologs (BUSCO) identifies COG genes from the assembled transcriptome to identify the orthologous core-genes present in the BUSCO pipeline [25]. BUSCO acts as an assessment of the transcriptome assembly by measuring the completeness of the transcriptome based on evolutionary present universal single-copy orthologs. The search revealed 87% of complete BUSCOS and 6% of fragmented BUSCOs indicating a near complete transcriptome for *C*. *lyallii*. Similarly, 64% of complete BUSCOs and 9% of fragmented BUSCOs were found in the case of *C*. *pallens*, suggesting a good transcriptome assembly [25].

### Functional annotation of the assembled unigenes

All the unigenes from both species were functionally annotated using the BLAST platform against characterised proteins from several species. By using a combined annotation from multiple species, the power of the annotation is increased and, consequently, provides a more accurate functional characterisation of the predicted peptides.

In total, 95,993 (70%) of the *C*. *lyallii* transcripts were annotated with 36,595 unique protein IDs. Most of the protein sequences were predicted by the NCBI-nr database. The greatest number of predicted homologous sequences belonged to sunflower followed by chrysanthemum, both belonging to the Asteraceae family. 90% of the translated amino acid sequences of the predicted transcripts were similar to the NCBI-nr database (ranging between 60% to 100% amino acid similarities). The predicted protein sequences were also BLAST searched against the proteome data from sunflower and chrysanthemum, separately. A total of 78,249 transcripts were annotated from the combined proteomic datasets of arabidopsis, sunflower and chrysanthemum. About 58% and 50% of the total predicted protein sequences in *C*. *lyallii* were successfully annotated from the two reference proteome datasets of sunflower and chrysanthemum, respectively, suggesting strong conservation of genetic networks between the members of the same family. BLAST searches against the uniprot database showed 53.89% similarity to the assembled *C*. *lyallii* transcriptome, with an E-value less than 1E-5.

A BLAST search against the NCBI-nr protein database yielded annotation of 160,482 (46%) transcripts present in the assembled draft transcriptome of *C*. *pallens*. Transcripts were also BLAST searched (using blastp) against the proteomic datasets of arabidopsis, rice and maize to obtain gene annotations. A total of 135,372 transcripts were annotated using the combined proteomic datasets. Most of the *C*. *pallens* transcripts (94,616) showed significant hits similar to the maize proteins with a shared 87% of average identity. Most of these transcripts have an E-value of less than 10^−6^ and more than 90% query coverage. About 42,235 and 89,299 unigenes were found to be significantly similar to arabidopsis and rice proteins, respectively. Similarly, 93,762 transcripts (E-value < 1E-5) showed on average 58% similarity against the uniprot database.

**Fig 1:**
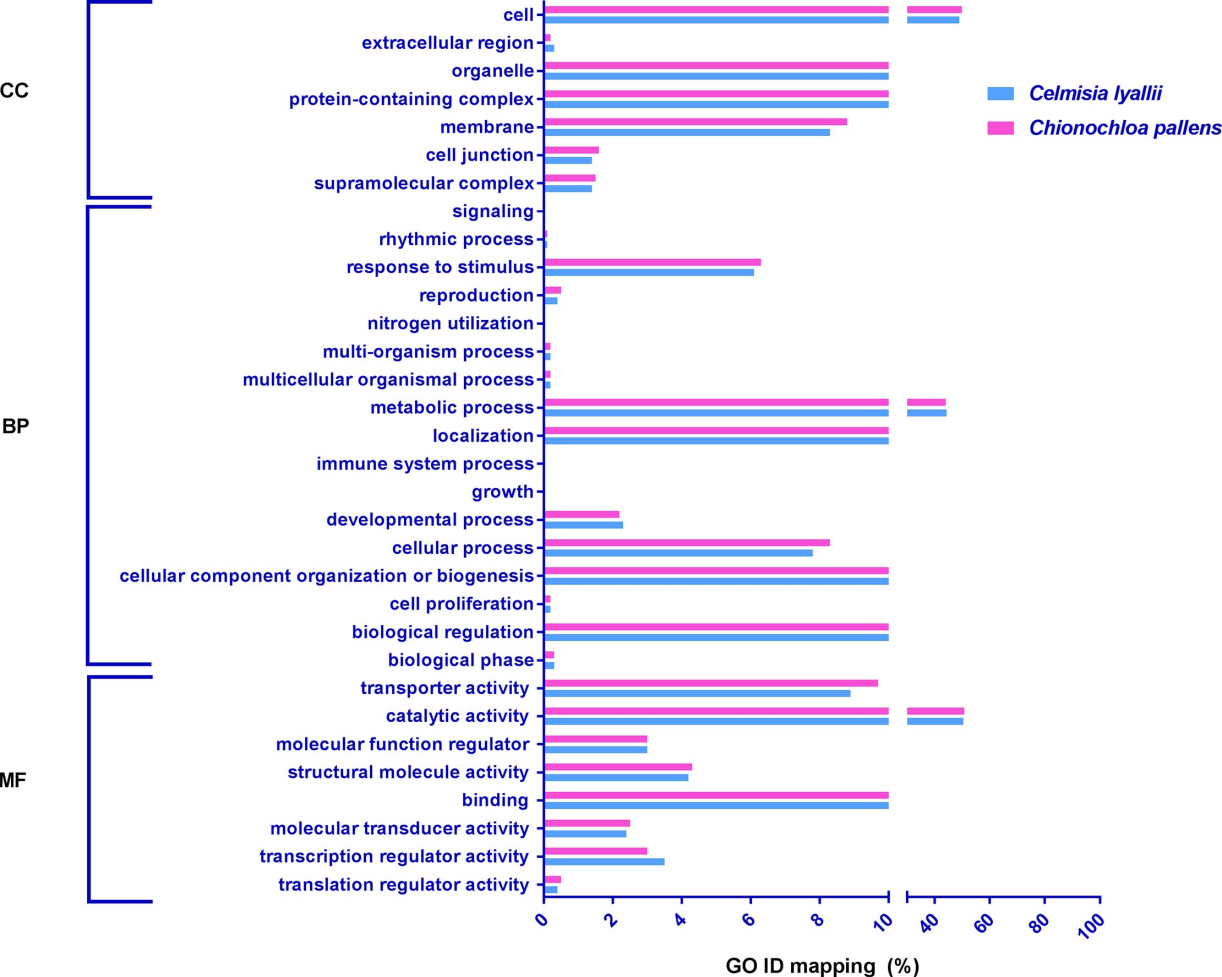
GO analysis of the assembled transcriptomes of *C*. *lyallii* and *C*. *pallens*. Transcripts were categorised into 32 different GO categories sub-divided into molecular function, biological process, and cellular component.

Each unigene in the assembled transcriptome was further categorised into three GO categories of ‘molecular function’, ‘biological process’, and ‘cellular component’ using PANTHER based on the sunflower and rice protein databases as references for *C*. *lyallii* and *C*. *pallens*, respectively. The assembled transcripts of *C*. *lyallii* and *C*. *pallens* were grouped into 32 different functional groups. A similar categorical pattern for functional grouping was observed for the annotated unigenes of both *C*. *lyallii* and *C*. *pallens*. Eight categories were found within the ‘molecular function’ subgroup, 18 within ‘biological processes’ and seven within ‘cellular component’ functional groupings for both *C*. *lyallii* and *C*. *pallens*. The top GO terms for ‘cellular component’ belonged to the cell component followed by the organelle functional group. For ‘biological processes’, the top GO terms were binding and catalytic activity. For ‘molecular function’, the top GO terms belonged to catalytic activity followed by binding and transporter activity (Fig 1). These functional groups are a reflection of current physiological responses and their regulation in the plants as they were growing under natural conditions.

### Transcription factor prediction

**Fig 2:**
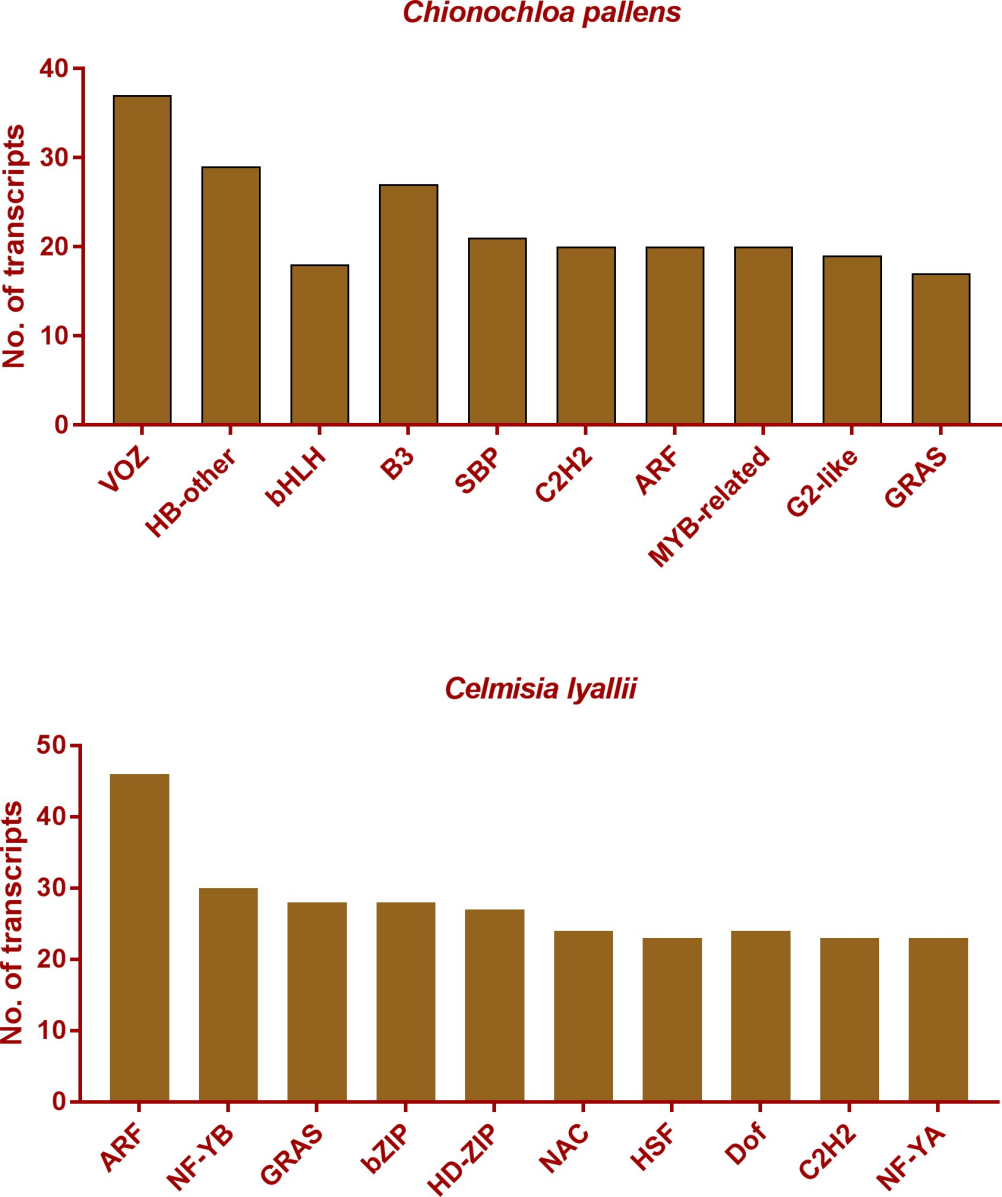
Identification of transcription factors from *C*. *lyallii* and *C*. *pallens*. A subset list of the number of transcription factors identified from the assembled transcriptome of *C*. *lyallii* and *C*. *pallens*.

Several transcription factor (TF) families, such as MADS-box and bHLH proteins, have been identified as key regulators of floral initiation and development. About 240 and 1696 unique transcription factors were identified from the assembled transcriptomes of *C*. *lyallii* and *C*. *pallens*, respectively (Fig 2).

Several MIKC^C^-type MADS box genes have been suggested to play crucial roles in flowering–time control. *FLOWERING LOCUS C* (*FLC*) has been shown to repress flowering and regulate the vegetative phase in both annual and perennial plants [37]. Exposing plants to cold (vernalisation) represses the expression of *FLC* and promotes flowering. *SUPPRESSOR OF CONSTANS 1* and *AGL-24* are two MADS-box genes that promote flowering under favourable conditions and support flower development [38]. Many of the MADS-box genes, including *SHORT VEGETATIVE PHASE (SVP)* and *MADS AFFECTING FLOWERING (MAF1/FLM*), have also been found to regulate the floral transition in response to ambient temperature change [39]. These transcription factors are good targets for temperature-assisted change in gene expression to modulate developmental phase transitions. In the present study, about 102 and 90 transcripts were found to be associated with MIKCc-type genes in *C*. *lyallii* and *C*. *pallens*, respectively (S2 Table). These gene(s) may play a key role in coordinating the vegetative to reproductive phase transition during masting.

In *C*. *lyallii*, the most abundant transcription factors were related to plant hormones and development, while in *C*. *pallens*, Vascular Plant One-Zinc-Finger (VOZ)-domain containing transcripts were present in abundance. VOZ transcription factors are involved in phytochrome B signalling. VOZ1 has been shown to downregulate *FLOWERING LOCUS C*, a floral repressor and to promote flowering in arabidopsis [40]. This suggests that the pathway of photoperiodic-mediated control of the flowering-time may be conserved in *C*. *pallens*. The other abundant transcription factors in *C*. *pallens* were also involved in plant growth and development processes.

### COG analysis and KEGG mapping

**Fig 3:**
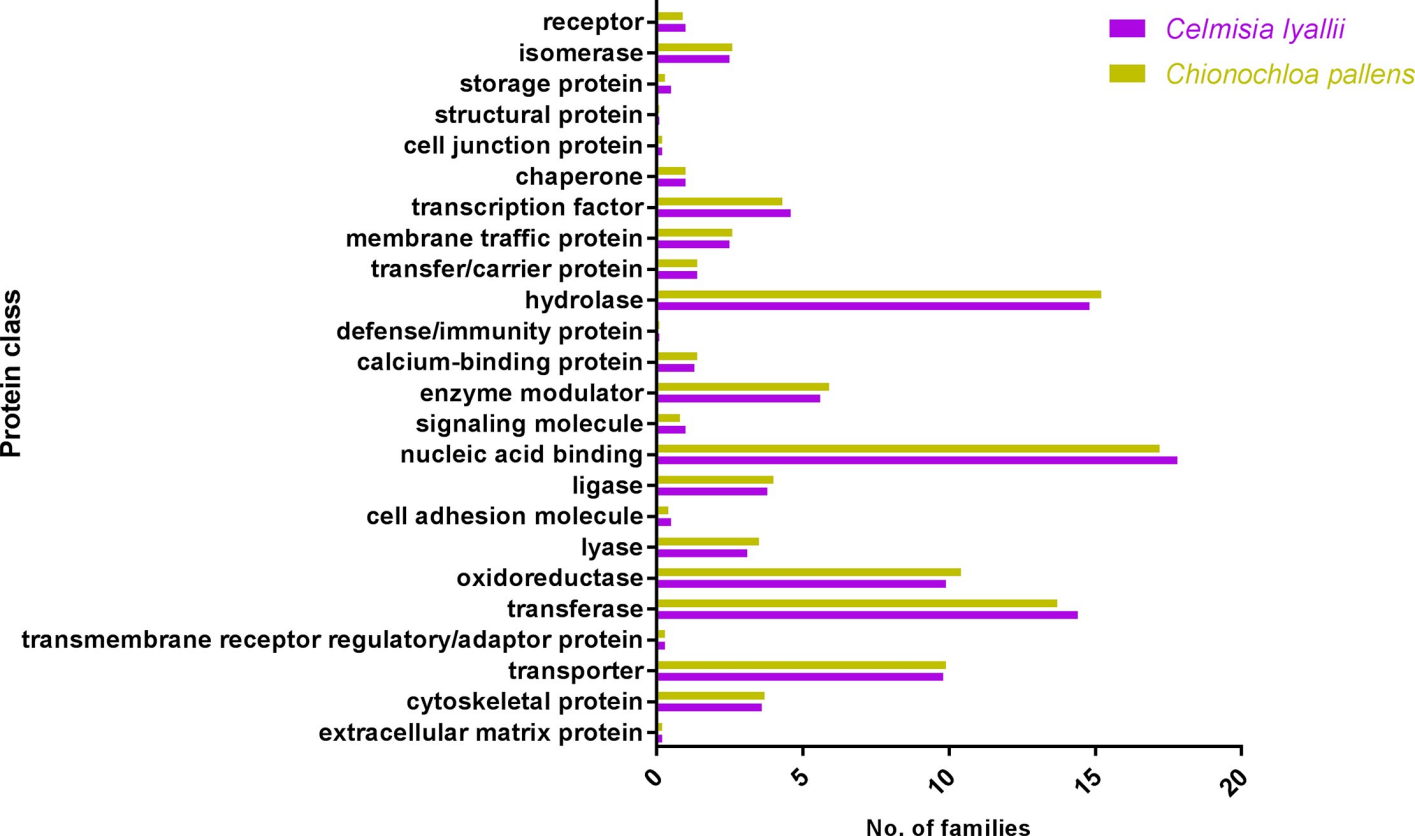
Identification of different protein classes. Categorisation of the peptide sequences predicted from the assembled transcriptome of *C*. *lyallii* and *C*. *pallens*.

**Fig 4:**
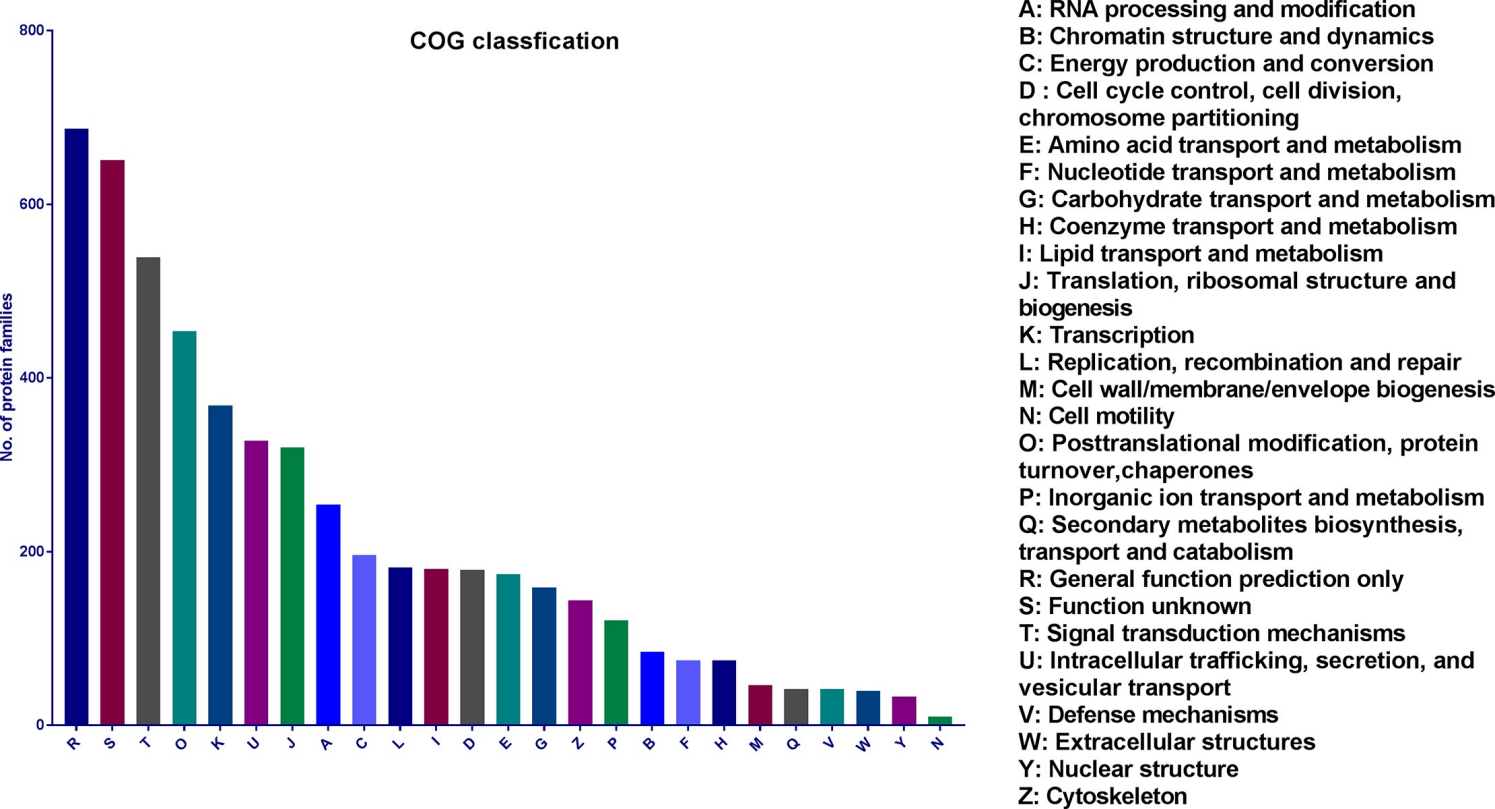
Functional classification of the *C*. *lyallii* transcriptome. The identified unigenes were further grouped into 25 categories based on the homology search against the COG database.

The assembled *C*. *lyallii* unigenes, annotated using reference proteins from sunflower, were further categorised into different protein classes (Fig 3). The majority of the transcripts were nucleic acid binding and hydrolase proteins, which is worth highlighting as most of the transcription factors involved in flowering are nucleic acid binding proteins [12]. The assembled unigenes were also searched against the COG database for functional characterisation. COG is a phylogenetic database comprised of protein sequences from 66 different genomes. These COGs are representative of conserved domains present in either individual proteins or paralogs from three distinct lineages. Most of the proteins belonged to the general functions category followed by unknown functions and signal transduction pathways (Fig 4). A very small fraction of the unigenes were found similar to the COG database (10.2%).

The predicted peptide sequences from the reference transcriptomes of both the species were also searched against the Pfam database for better annotation. About 2485 transcripts from the *C*. *lyallii* reference transcriptome were found to be similar to known protein families in the Pfam database with ‘PRR repeat family’(19.3%), ‘Protein kinase domain’ (14.9%), ‘Protein tyrosine kinase domain’(9.8%) and ‘WD domain’(5.79%), ‘RNA recognition motif’ (5.35%) as the top five categories. In the case of *C*. *pallens*, 967 transcripts were predicted when aligned to the database with Protein Kinase domain’ (14.06%), ‘Protein tyrosine kinase’ (8.7%), ‘WD-domain’ (7.75%), ‘RNA recognition motif’ (5.99%), and ‘Leucine rich repeat’ (5.17%) as the top five protein families identified. Unigenes were then mapped to KEGG pathways by using the translated peptide sequences using arabidopsis as a reference for pathway analysis. 26.8% of the *C*. *lyallii* and 28.7% of the *C*. *pallens* unigenes mapped to EC numbers in 119 KEGG pathways. The largest number of unigenes were mapped to metabolic pathways (11% in *C*. *lyallii* and 12.4% in *C*. *pallens*) followed by secondary metabolite synthesis (6.2% in *C*. *lyallii* and 6.8% in *C*. *pallens*).

### Identification of flowering-time genes in *Celmisia lyallii* and *Chionochloa pallens*

There are over 300 flowering-time genes identified in arabidopsis responsible for the transition to flowering (29). Expression analysis of these flowering-time genes may help unravel the mechanism of floral transition in masting plants using differential temperature as a cue [17]. Previously characterised flowering-time genes from arabidopsis and rice were used as references to identify the corresponding homologous sequences in *C*. *lyallii* and *C*. *pallens*, respectively. The assembled transcriptomes were imported and maintained in the form of a collective database at the CLC Genomics Workbench 8. To validate and annotate the assembled unigenes, gene sequences from arabidopsis and sunflower were used as the reference sequences for *C*. *lyallii*. Although the core genes accountable for regulation of flowering are similar in both monocots and dicots, the flowering regulatory network consisting of temperature and photoperiodic sensing genes are different in monocots compared to dicots. Therefore, protein sequences from rice and maize were used as references for flowering gene(s) identification in *C*. *pallens*, as they both belong to the Poaceae family. Using blastp from the NCBI BLAST suit, floral gene sequences from the draft transcriptomes of *C*. *lyallii* and *C*. *pallens* were identified under stringent selection criteria of E-value of less than 1E-3, 70% protein identity and 50% query coverage. Such criteria should eliminate the risk of improper identification of homologous sequences due to phylogenetic distances and ploidy level of the genome. In total, 543 and 470 transcripts, matching with 97 and 110 unique floral protein IDs were identified in *C*. *lyallii* and *C*. *pallens*, respectively (S2 Table).

As mentioned in previous reports, ecological transcriptomic data can aid in development of new genomic resources for identification and characterisation of genes involved in floral transition and development in masting plants. Huang et al. (2013) identified 27 potential floral transcription factors such as *FLC* and flower development genes in hickory using transcriptomic data [41]. Photoperiodic genes including *CONSTANS*, *GIGANTEA* and *PHYTOCHROME B* are regulated by the circadian rhythm in plants and, subsequently, control the floral transition in response to changes in daylength [42], while temperature-mediated floral transition is modulated by genes including *SVP* and *APETELA2* (*AP2*) which can repress flowering at lower temperatures under inductive conditions [43]. Identification and seasonal analysis of similar homologous target genes can provide significant insight into regulation of flowering in masting plants.

### Tissue-specific expression of flowering-time genes in *Chionochloa pallens*

**Fig 5:**
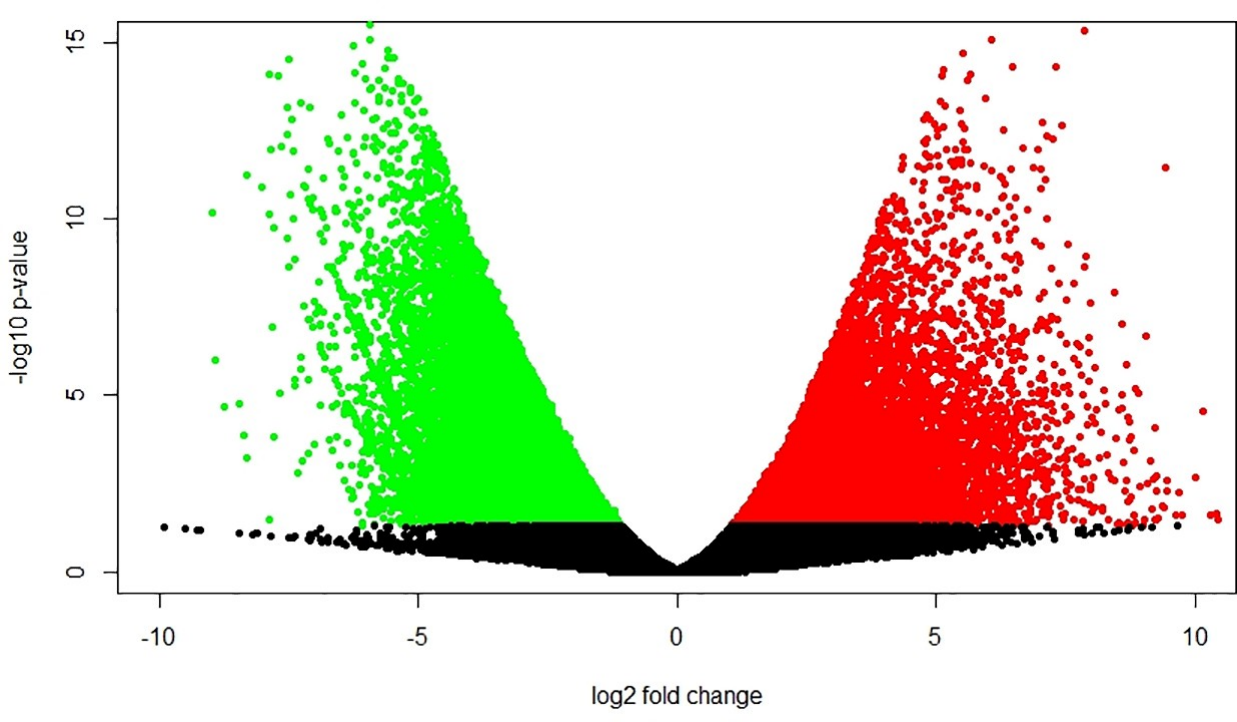
Volcano plot showing differentially expressed genes. 15,234 significantly differentially expressed genes were identified between the leaves and apical meristems. The red dots represent significantly up-regulated genes and green dots represent significantly downregulated genes with a P-value < 0.05.

**Fig 6:**
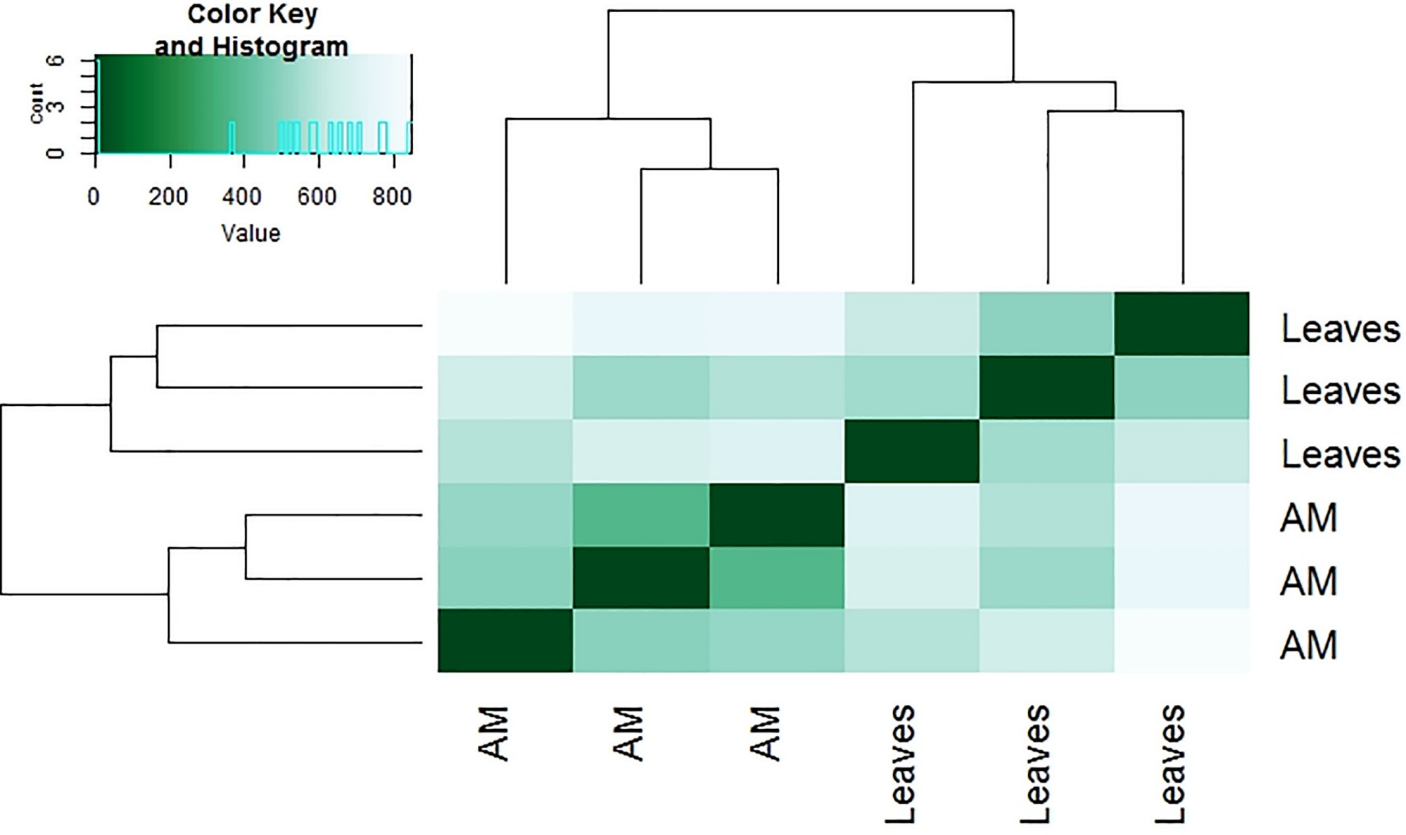
Comparisons of transcriptional profiles across samples. Heat-map showing hierarchical clustering resulting from a pairwise comparison of transcript expression levels. Clustering is represented between three separate biological replicates of leaves and apical meristematic (AM) tissue, respectively.

Pairwise comparisons were used to analyse the differentially expressed genes between leaves and apical meristematic tissues of *C*. *pallens*. Using a significance threshold of 0.005 False Discovery Rate and 2-fold change in expression, we determined that there were 15,324 differentially expressed genes between the two tissues (Fig 5). Transcripts showed distinct expression patterns as they emerged into two separate groups from leaves and apical meristems respectively (Fig 6). There were 124 differentially expressed transcripts belonging to 38 unique floral genes in the leaves compared to the meristematic tissue.

**Fig 7:**
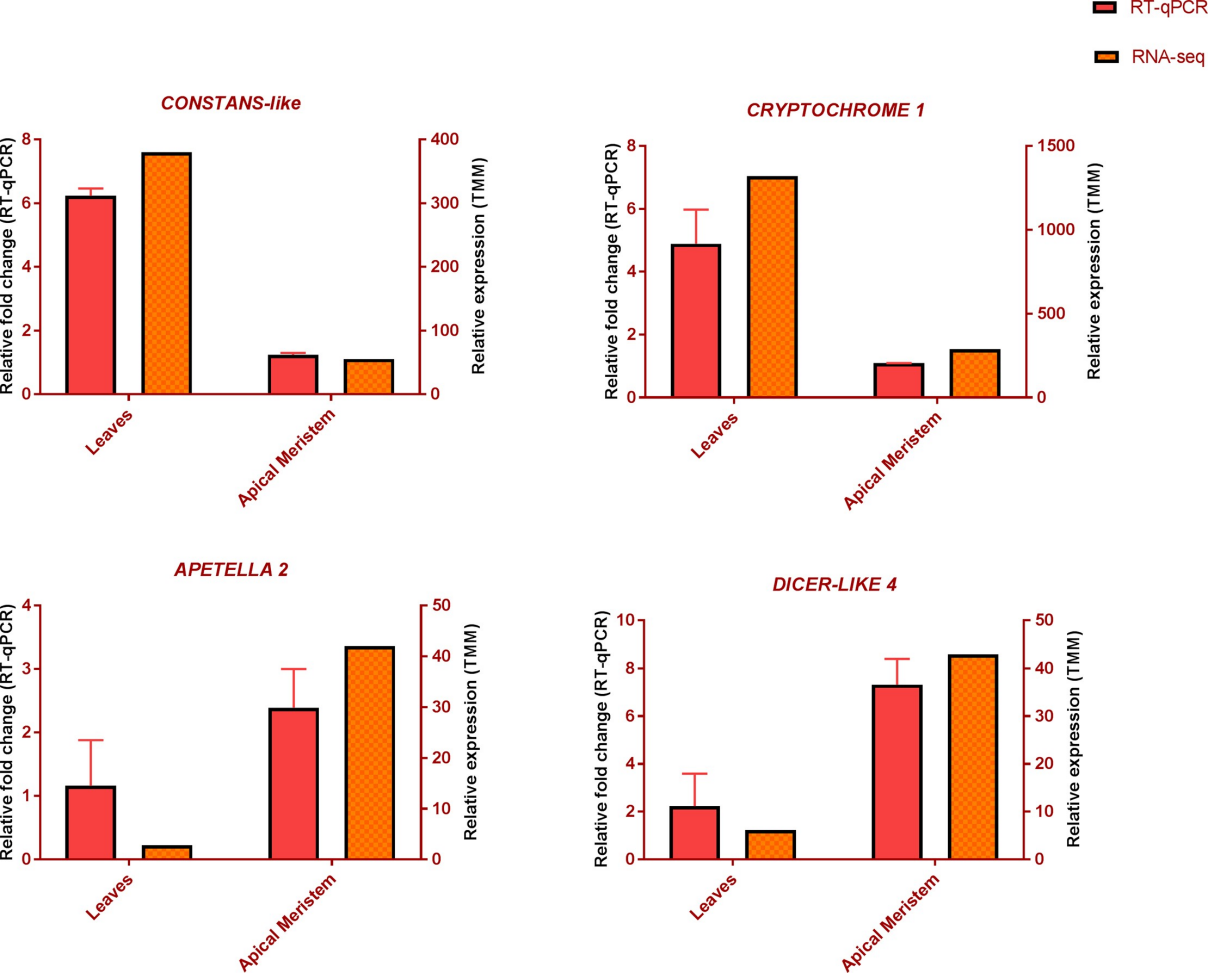
Relative expression of the flowering-pathway genes *CONSTANS-LIKE*, *CRYPTOCHROME*, *APETELA 2*, and *DICER-LIKE 4* across leaves and apical meristems. Expression profile is based on RNA-seq data, normalised by the TMM method and RT-qPCR data showing relative fold change, normalised to the selected reference genes. The RT-qPCR experiment was performed using three biological replicates for each tissue with three technical replicates ±S.D.

Flowering is a complex molecular process regulated by external and endogenous cues. Several activators and repressors of flowering have been characterised in arabidopsis and other plant species controlling flowering time [38]. In the differential gene expression analysis, most of the floral activators including *SPL9* and *SPL3* were strongly downregulated in the leaf tissue. These downregulated genes are involved in regulation of temperature, hormonal, and age-mediated flowering time control. Expression of many epigenetic modifiers responsive to temperature changes were also downregulated. Photoperiodic genes such as *CONSTANS*, *CRYPTOCHROME1* and *PIF4* and sugar signalling genes were upregulated in the leaves (Fig 7). Changes in the expression of epigenetic modification genes and photoperiodic genes can be correlated with the long days during the summer when the samples were collected. *CONSTANS* (*CO*) and *CRYTOCHROME1* (*CRY1*) are two strong floral promoters which activate FT under inductive conditions [44]. PIF4 integrates multiple environmental and endogenous cues including photoperiod, temperature, sugar and gibberellin levels [45]. Kumar at al. (2010) showed that the PIF4 protein in arabidopsis acts as a thermosensor to promote flowering at high ambient temperature [46–48]. This initial analysis suggests that the core components of the flowering network are conserved in *C*. *pallens* and could be involved in activation of flowering during a masting event. The study also provides evidence of a role for floral epigenetic modifiers which may play a crucial role in temperature-mediated synchronised flowering [48].

### Seasonal expression of the flowering-time genes identified in *C*. *lyallii* and *C*. *pallens*

**Fig 8:**
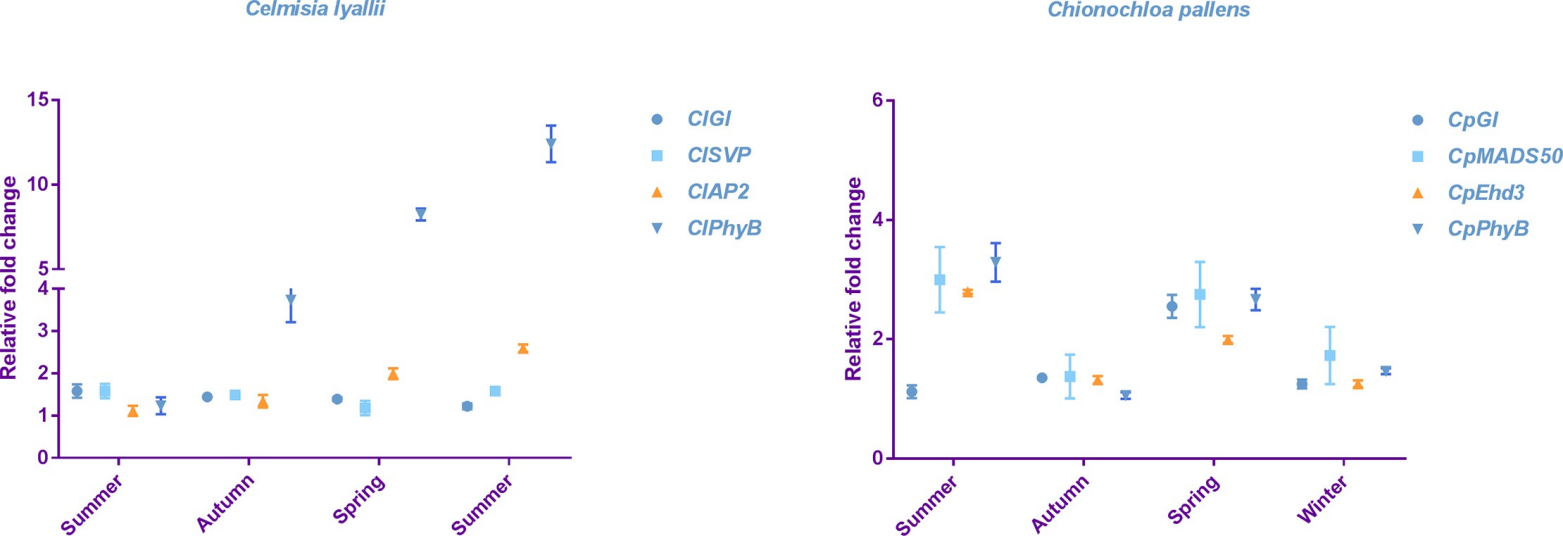
Seasonal gene expression analysis of selected flowering-pathway genes in *C*. *lyallii* and *C*. *pallens*. The relative expression was calculated using 2^-ΔΔCt^ method, represented by data from two biological replicates with three technical replicates ± S.D.

Expression of the key flowering time genes, based on the literature and their presence in the RNAseq data, was monitored to determine their potential role in the growth and development of the two masting plants (Table 2). Due to destructive sampling (collection of leaves and apical meristems), it was not possible to predict whether the leaves subtended meristems that would have flowered and draw conclusions on the molecular network of flowering. Hence, leaves from both species were sampled throughout the year, representing four different seasons (summer, autumn, spring, summer for *C*. *lyallii* and summer, autumn, spring, winter for *C*. *pallens*) (Fig 8). Transcriptomic analysis suggested a strong involvement of photoperiodic response regulators of flowering under the long days of the summer season. Based on the literature search, several of the photoperiodic homologous genes identified from the reference transcriptome of *C*. *lyallii* and *C*. *pallens*, and which are also regulated by temperature, were selected for expression analysis. Expression analysis of these selected genes was carried out using RT-qPCR with two biological replicates, each of which comprised a combined pool of three individual plants.

**Table 2:** Annotation of flowering-related genes selected for RT-qPCR analysis.

***C*. *lyallii***							
**Gene**	Gene name	Arabidopsis locus ID	Sunflower locus ID	% Identity	E-value	%Gaps	Contig ID
*ClGI*	GIGANTEA	AT1G22770	HQ428760.1	78.53	0	1.75	TRINITY_DN48665_c1_g2_i1
*ClPHYB*	PHYTOCHROME B	AT2G18790.1	GU985581.1	86.57	0	0	TRINITY_DN47433_c0_g1_i1
*ClAP2*	APETALA2	AT4G36920	XP_021998113	81.65	1.90E-75	0	TRINITY_DN45405_c1_g1_i4
*ClSVP*	SHORT VEGETATIVE PHASE	AT2G22540	XP_022001248	85.27	2.66E-96	0.45	TRINITY_DN40059_c1_g1_i3
***C*. *pallens***							
Gene	Gene name	Arabidopsis locus ID	Rice locus ID	% Identity	E-value	%Gaps	Contig ID
*CpGI*	GIGANTEA	AT1G22770	CAB56058.1	95.24	0	0	TRINITY_DN35492_c2_g6_i1
*CpPhyB*	PHYTOCHROME B	AT2G18790.1	CAA40795.2	90.96	0	0	TRINITY_DN28349_c2_g6_i1
*CpEhd3*	EARLY HEADING DATE 3		BAI77463.1	78.35	3.27E-52	0	TRINITY_DN32679_c1_g8_i1
*CpMADS50*	SUPPRESSOR OF OVEREXPRESSION OF CONSTANS 1	AT2G45660	Q9XJ60.1	78.39	2.40E-105	1.4	TRINITY_DN25163_c1_g3_i4

In *C*. *lyallii*, homologs of *GIGANTEA* (*GI*), *PHYTOCHROME B* (*PhyB)*, *SHORT VEGETATIVE PHASE* (*SVP*), and *APETELA 2* (*AP2*) were selected for seasonal expression analysis. GI and PhyB are two crucial proteins regulating circadian rhythm- and photoperiodic-mediated flowering control [44]. GI acts as a floral promoter by stabilising the protein CONSTANS which then activates FT, whereas PhyB forces CO to undergo proteolysis and thus represses its activity during the day [42]. *SVP* and *AP2* are regulators of age and maturity of the plant and are repressors of flowering [49]. All the selected genes showed significant seasonal variation (Fig 8). Expression of *ClGI* was greatest during summer, followed by a decrease in the late autumn and winter (*P-*value <0.001). At the same time, the expression of *ClPhyB* was low during the summer but increased sharply in the following seasons. The expression of *ClPhyB* was seven times greater in the early spring samples compared to the previous summer season (*P-*value <0.001). The expression pattern of *ClAP2* was similar to *ClPhyB*, with greater expression in the following spring and late summer compared to the previous summer. *ClSVP* had greater expression in the samples collected in summer (Fig 8). The greater expression of *ClSVP* during the summer season may interfere with the perception and signalling activation of the floral promoting genes [50]. This analysis suggests that *ClPhyB*, *ClSVP* and *ClAP2* may be responsible for blocking the activation of the floral transition in *C*. *lyallii* (F = 106.5, 9; *P-*value <0.001).

Homologs of *GI*, *PhyB*, *MADS50* and *EARLY HEADING DATE 3* (*Ehd3*) genes were selected for initial expression analysis in *C*. *pallens* leaf samples collected over the year. *MADS50* and *Ehd3* are the activators of floral promoting genes in response to temperature and day length [51, 52]. Expression of *CpGI* was found to be greater in the early spring samples compared to the other samples (*P-*value <0.003). While there were differences in the seasonal expression pattern of *CpPhyB*, *CpEhd3* and *CpMADS50*, all of these genes had a greater expression in the summer season compared to the other seasons (F = 50.26, 3, *P-*value <0.001). There was a decrease in the expression of these genes in late autumn followed by an increase in the spring and a drop in the winter. (Fig 8). Previous expression studies in rice [51] and maize [53] growing in lab conditions also showed seasonal expression profiles for *Ehd3* and *PhyB*, respectively. The RNA-seq allowed us to identify flowering-related genes for both species, and RT-qPCR enabled us to verify their seasonal expression patterns. High summer temperatures are known to activate the floral transition in *C*. *lyallii* and *C*. *pallens*. However, no significant difference was observed in the expression of genes that either promote or repress flowering during the summer season. Thus, the absence of a potential activation signal of flowering could explain why none of the plants flowered in the next season.

The results indicate that there is another level of regulatory network in *C*. *lyallii* and *C*. *pallens* controlling the floral transition beyond a relatively few floral promoters or repressors. Even though *C*. *lyallii* and *C*. *pallens* belong to separate plant families, most of the floral genes identified in arabidopsis were also shown to be conserved in these non-model species. The core components of calcium signalling, photoperiodic signalling and thermosensory pathway genes regulating the floral transition were found in both species. Our transcriptome data open new possibilities of assessing the transcriptome profile of several other candidate genes possibly related to the flowering process of *C*. *lyallii* and *C*. *pallens*, including temperature-responsive floral regulators [48], and crucial floral genes [49], such as *FT*, *FLC*, *TFL1* and *VRN1*. It will also be interesting to assess the transcriptomic profiles of other temperature-responsive floral regulators [48] during induction of masting. Global expression profiling of floral and epigenetic regulators can, therefore, improve our knowledge of the cause of irregular patterns of floral development in perennial plants.

## Conclusion

Ecological transcriptomics is the foundation upon which our investigation into the underlying molecular mechanism controlling mast flowering is based. This new era of molecular biology is based on the extensive advances in next-generation sequencing analysis where, with access to specialised sequencing technologies, we can ask significant ecological questions relating to plants growing under natural conditions. Due to the large genomes and high ploidy levels of perennial plants, transcriptomics stands out as an exceptional alternative for gene identification instead of whole-genome sequencing. In this work, high-quality RNA-seq was used to identify and study global flowering gene expression patterns seasonally and between different tissues of a masting plant. These initial flowering-time expression analyses established the conservation of the flowering network in our masting plants. Our study also indicated a role for changing seasonal conditions, such as temperature or day length, in controlling the floral transition in the masting plants. In the future, our molecular phenological study will help unravel the genetic and molecular mechanisms involved in the regulation of genes related to the masting process and explain why plants have adopted this mode of delayed reproduction. This study stands as a prime example of the use of ecological transcriptomes to understand a significant ecological phenomenon.

## Supporting information

S1 Fig Number of RNA-seq studies published in the field of plant sciences from 2010–2019. (PDF)

S1 Table List of primer sequences used in the present study. (PDF)

S2 Table List of flowering genes identified in *Celmisia lyallii* and *Chionochloa pallens* with their similarity search values and List of Pfam annotated transcripts. (XLSX)
