# Supplementary figures and images for: Identification of flowering-time genes in mast flowering plants using De Novo transcriptomic analysis
Source: PLoS One. 2019 Aug 14;14(8):e0216267. doi: 10.1371/journal.pone.0216267 (PMC6693765; doi:10.1371/journal.pone.0216267)

# S1 Fig: RNA-seq studies in the field of plant sciences

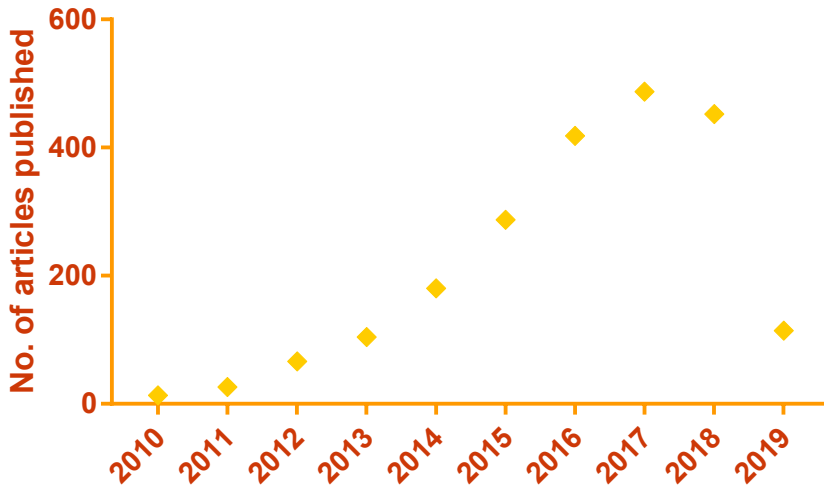

Supplement: S1 Fig — (PDF) [file pone.0216267.s001.pdf]
