# Supplementary material for: Identification of flowering-time genes in mast flowering plants using De Novo transcriptomic analysis
Source: PLoS One. 2019 Aug 14;14(8):e0216267. doi: 10.1371/journal.pone.0216267 (PMC6693765; doi:10.1371/journal.pone.0216267)
Supplement: S1 Table — (PDF) [file pone.0216267.s002.pdf]

S1 Table: List of primer sequences used in this study for Celmisia

**Primer name**	Forward primer sequence	Reverse primer sequence	Size of amplicon	Tm (ºC)	GC %
PP2A	5’-CCAACGCTGCATGGTTCCTCT-3’	5’-GGTTCCAGTGAGCCTGAATGTTC-3’	191	60	57.1
GAPDH	5’-ATACTTTGTCGTCATCGTCATCTTCAC-3’	5’-CCTGGTCGGTGGATATTGTTGTAGA-3’	202	58	44.3
GI	5’-TGTTGCTAATGGTGCTGGTGTCA-3’	5’-GGCTGTTGTTGGAGGAGGAAGTAA-3’	108	60	47.8
AP2	5’-CAATCTGACCAAGGAGGAGTT-3’	5’-CCGAAGTCGCATTGTTACC-3’	220	58	45.2
SVP	5’-GCTGTTATTCTCTTCTCTTCCAATG-3’	5’-CCTCACCTCTCAACTGCCTTA-3’	214	58	40
PhyB	5’-TCGTGCGTTAAGAGGTGAAGAAGA-3’	5’-CGAAGTTCCACTCTGAGCAACATG-3’	296	58	47

S1Table: List of primer sequences used in this study for *Chionochloa*					
**Primer name**	Forward primer sequence	Reverse primer sequence	Size of amplicon	Tm (ºC)	GC%
ExP	5’-GCACCACCTCTGAAGCCAAG-3’	5’-CCACATATAAGACAACCAGTCATCG-3’	213	58	1.968
THP	5’-AGAAGGAACTTGGTGGCAGACTC-3’	5’-TCCGTGAAGGTGGTTGACATTGT-3’	176	60	2.045
GI	5’-GCTGAACTCCGCACAATGAT-3’	5’-GCTCGCAAGATAAGGCACAA-3’	280	58	50
MADS50	5’-GCAGTCGTCACCGTGGAAGAT-3’	5’-AGCCTTGTTGGAGCGATGTTCT-3’	138	60	46
Ehd3	5’-ATA AGCCTGATGAGGTGATTGG-3’	5’-CTCGTGAAATGACTGAAAGACTG-3’	174	60	44.6
PhyB	5’-GCTTCTTCCTCATCGTCCTT-3’	5’-TACAACAACAGCGTCCACAT-3’	191	60	45.2
